# Forecasting the Major Influences of Predation and Environment on Cod Recovery in the Northern Gulf of St. Lawrence

**DOI:** 10.1371/journal.pone.0082836

**Published:** 2014-02-11

**Authors:** Nicolas Bousquet, Emmanuel Chassot, Daniel E. Duplisea, Mike O. Hammill

**Affiliations:** 1 Institut de Mathématiques de Toulouse, UMR 5219 CNRS, Université Paul Sabatier, Toulouse, France; 2 Institut de Recherche pour le Développement, UMR 212 EME (IRD/IFREMER/UM2), CRH, France & Seychelles Fishing Authority, Victoria, Seychelles Island; 3 Fisheries and Oceans Canada, Mont-Joli, Québec Canada; University of Catania, Italy

## Abstract

The northern Gulf of St. Lawrence (NGSL) stock of Atlantic cod (*Gadus morhua*), historically the second largest cod population in the Western Atlantic, has known a severe collapse during the early 1990 s and is currently considered as endangered by the Committee on the Status of Endangered Wildlife in Canada. As for many fish populations over the world which are currently being heavily exploited or overfished, urgent management actions in the form of recovery plans are needed for restoring this stock to sustainable levels. Stochastic projections based on a statistical population model incorporating predation were conducted over a period of 30 years (2010–2040) to assess the expected outcomes of alternative fishing strategies on the stock recovery under different scenarios of harp seal (*Pagophilus groenlandicus*) abundance and environmental conditions. This sensitivity study shows that water temperature is key in the rebuilding of the NGSL cod stock. Model projections suggest that maintaining the current management practice under cooler water temperatures is likely to maintain the species in an endangered status. Under current or warmer conditions in the Gulf of St. Lawrence, partial recovery might only be achieved by significant reductions in both fishing and predation pressure. In the medium-term, a management strategy that reduces catch could be favoured over a complete moratorium so as to minimize socio-economic impacts on the industry.

## Introduction

The global expansion of world fisheries has sequentially led to the intense and/or over-exploitation of the majority of world's major fish stocks, with only 1% undergoing some form of recovery from depletion [1]–[4]. Signatories of the 2002 World Summit on Sustainable Development have however committed to maintain or restore fish stocks to levels providing Maximum Sustainable Yield (MSY) by 2015. From a global perspective, rebuilding depleted marine resources and fisheries will require a substantial reduction in exploitation rates. This can be achieved through reductions in catch quotas potentially combined with spatial and technological management measures [5], [6]. The complexity of population dynamics responses to management actions at low population sizes has been highlighted, through past fishery experiences, by the ability of stocks to rebuild to former levels of abundance [5], [7]–[9]. Processes such as depensation, higher demographic stochasticity, and potential genetic changes in vital rates might affect the recovery of depleted stocks [10]–[12]. In addition, multispecies interactions, predator pits (i.e., where predation probability decreases above and below an intermediate level of the prey abundance), as well as oceanographic and environmental conditions have been shown to affect the productivity and rate of recovery for some fish populations [5], [13].

**Figure 1:**
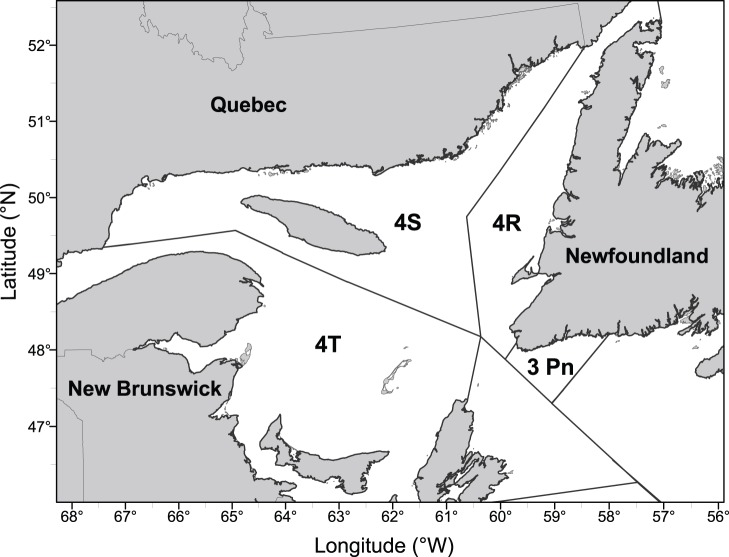
Northern Gulf of Saint Lawrence (NAFO divisions 4R and 4S).

During the early 1990 s, there was an almost simultaneous collapse of most of the Atlantic cod (*Gadus morhua* L.) stocks in Canada, leading to severe reductions in quotas and temporary moratoria on commercial fishing [14], [15]. Despite these restrictive management measures, Canadian cod populations have remained at low abundances for more than a decade. Several interrelated factors have been put forward to explain the lack of recovery of cod stocks, including recruitment failure, high fishing mortality, elevated natural mortality, and poor fish condition [16], [17]. The northern Gulf of St. Lawrence (NGSL) cod stock (NAFO divisions 3 Pn4RS; Figure 1) was historically the second largest cod population in the Western Atlantic, supporting a fishery of more than 100,000 t in 1983 [18]. By the late 1980 s, the population and fishery had gone into such a decline that two successive moratoria were imposed during 1994–1996 and 2003. Nonetheless, today the stock biomass remains below safe biological limits. In April 2010, the Committee on the Status of Endangered Wildlife in Canada (COSEWIC) re-examined the NGSL cod stock and recommended its status be changed from ‘threatened’ to ‘endangered’, suggesting that the stock would face imminent extirpation.

The current lack of recovery is likely due to a combination of poor productivity of the NGSL cod stock associated with ongoing fishing activity that removes most of the stock's surplus production [17], [19], [20]. The condition of individual fish has been suggested as a useful proxy for monitoring seasonal changes in energy available for the different processes influencing cod productivity [21]. Fish condition has also been shown to be a good integrative indicator of the energy content of cod, which may affect mortality rates and be important to reproductive capacity [21]–[24]. Variations in growth and condition have also been shown to reflect changes in environmental conditions and prey availability [25]–[29]. For instance, the coincidence of smaller length-at-age and poor condition suggested that cod and/or their prey were experiencing unfavourable environmental conditions during the period of stock collapse, having little energy reserves to survive over the winter months or critical stages of their life cycle [21], [26]. The cold intermediate layer (CIL) is a prominent feature of the temperature and salinity structure of the Gulf of St. Lawrence [30]. In the NGSL, the below-normal temperatures of the CIL observed over the mid-1980 s to the late 1990 s could explain the poor survival and condition of cod during the period of stock collapse [19]. Changes in the CIL temperature might also affect the early and adult stages of cod by modifying their migration and distribution patterns [31].

In addition to fishing and environmentally-driven condition, higher natural mortality resulting from increased predation has been proposed as a plausible hypothesis explaining the collapse and failure of Northwest Atlantic groundfish populations to recover [16], [32]–[34]. In the southern Gulf of St. Lawrence, elevated mortality amongst large cod, possibly due to grey seal (*Halichoerus grypus*) predation may be limiting recovery of that cod stock [34], [35]. In the NGSL, harp seal (*Pagophilus groenlandicus*) predation might play a significant role in the failure of the cod stock to recover [33], [36]. Harp seals are currently at their highest abundance in over 50 years, with a mean population of about 8.2 million animals during 2008–2010 [37]. Although there is a commercial harvest and the current Total Allowable Catch (TAC) is 400,000, recent catches have been less than 100,000 per year [38]. A reduction in seal numbers may promote NGSL cod rebuilding. However, removing top predators from ecosystems might lead to unexpected effects on prey and competitor populations as well as fisheries due to the high connectivity and complexity of marine food webs [39]–[41].

The status of ‘endangered’ for the NGSL cod calls for an urgent and strong recovery plan for the stock. Modelling population trajectories in the face of uncertainties in the current stock abundance, recruitment, and mortality rates could provide information on the best approach to promote recovery. An age-structured model was recently developed to include the different sources of mortality that affect the NGSL cod stock [42], [43]. In the model, changes in fish condition, used as a proxy of environmental conditions, affect growth, recruitment, and natural mortality. Model results showed that the collapse of the NGSL cod stock was mainly due to the combination of high fishing mortality rates and poor environmental conditions in the early to mid-1990 s contributing to the current state of recruitment overfishing.

In the present analysis, the Seal IMpact on Cod Abundance (SIMCAB) model was used to run long-term stochastic projections on the effects of environmental conditions, seal predation, and fishing on the recovery of the NGSL cod stock. Following an estimation procedure based on survey and commercial data between 1984 and 2009, several prospective scenarios were considered to cover a large range of conditions that could be experienced by cod in the future and affect the recovery of the stock. Different levels of reduction in seal numbers were simulated in accordance with recent reference points defined for the harp seal population of the Northwest Atlantic [44]. Harvest control rules (HCRs) are the operational procedures of a fisheries management policy that determine the annual fish catch quotas as a function of stock status. Alternative HCRs based on a conservative approach (i.e. moratorium), a reduced catch approach allowing fishing (roughly at 50% of current levels), and on the current way of allocating total allowable catch (TACs) in the NGSL fishery were used for the projections. Projection scenarios were conducted considering alternative temperature regimes for the cold intermediate layer (CIL) of the Gulf of St. Lawrence.

The objectives of the present analysis were (i) to assess the major expected effects of a decline in seal numbers on the recovery of the NGSL cod stock considering alternative ecosystem states and harvest control rules, (ii) to estimate the time-span required for rebuilding the spawning stock biomass above limit reference points, and (iii) to identify the most suitable strategy to secure the NGSL stock from recruitment overfishing and escape the COSEWIC status of ‘endangered’.

## Materials

### Harp Seal Abundance Data

Abundance of the Northwest Atlantic harp seal population was estimated for the period 1952–2010 based on an age-structured model fitted to pup production data and incorporating information on reproductive rates, reported removals, as well as estimates of non-reported removals and losses through bycatch in other fisheries [37]. As in Chassot et al. (2009), only a fraction between 25% and 33% of seals, that move into the Gulf each year, is considered for the assessment (Figure 1 in File SIM).

### Cod Relative Abundance and Catch Data

Stratified, random bottom trawl surveys have been conducted by the Department of Fisheries and Oceans Canada in the Gulf of Saint Lawrence annually, in summer months, since 1984. A consistent time series of numbers-at-age, accounting for the changes in research vessels and fishing gear, was used as abundance indices for cod for the period 1984–2009 [18], [34]. The catch-at-age matrix of cod (number of individuals) was obtained from the stock assessment carried out in February 2011 and included quantities landed for both commercial and recreational fisheries, excluding discards.

### Cod Biological Data

Sex ratio and maturity ogive data were derived from winter surveys conducted with the MV “Gadus Atlantica” from 1984 to 1994 and spring samples available from the Groundfish Sentinel Fisheries Program thereafter (www.osl.gc.ca./pse/en/). Fulton's condition factor [45] based on length and weight data was obtained from the winter surveys (1984–1994), the Sentinel Fisheries Program (1995–2010), and supplementary research surveys conducted during the pre-spawning period in 1994, 1995, 1997, and 1998 [20], [46], [47].

## Methods

This section describes the model and the successive steps adopted in the present analysis: (i) estimation, (ii) validation, and (iii) projection. The main features of the model are explained in the two following subsections, and further details can be found in the Supporting Information Material (File SIM). SIMCAB is not a predator-prey model, since it does not incorporate feedback effects on seal population attributable to the variations of the resource, as usual in such models. Especially in the projection study, it is hypothesized that those effects remain moderate because harp seals are generalist predators characterized by a diverse diet of fish and invertebrates [48], [49], which limits the sensitivity of seal abundance to the state of the cod stock. This assumption is later discussed in the final section of the article.

First, a maximum likelihood estimation of the unknown parameters was performed by fitting the model to the 1984–2009 time period. Second, a validation procedure was conducted by leading a retrospective analysis between 2005 and 2009 (cf. File SIM). Adding the effects of environmental noise, a stochastic version of the model was then produced to conduct projections. The relevance of this simulation model was statistically tested as follows. The retrospective estimations made between 2005 and 2008 defined four simulation models that projected cod abundances between 2006 and 2009. For coherence reasons, the projection were constrained by the observed catches and mean water temperatures between these years, namely the cod conditions were resampled using the noisy linear relation established before (Equation (1) in File SIM) and the observed CIL temperatures (Figure 10 in File SIM). The retrospective maximum likelihood estimates of survey catchability (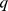), selectivity-at-age (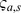), and observation variance (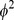) were used to produce a series of predictive distributions 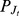 of annual survey indices 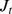 (Equation E14 in Table 1) which could be compared to observations 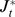. Observing a suprising (i.e. extreme) 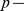
value 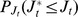 would imply that the observation 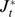 is unlikely under the regime of the projection model, which would invalidate the modelling approach. A similar validation study was conducted over observed total catches 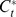 and proportions-at-age for catches and survey indexes. Finally, population projections were conducted with a stochastic version of the model including environmental noise, during 2010–2040, under different scenarios of seal predation, environmental conditions, and fishing strategies, and accounting for constraints in 2010 known at the time of the study (e.g., commercial catches).

**Table 1:** Parameters, variables and associated equations used in the SIMCAB estimation model. 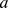 and 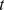 index age and year, respectively. NoI: number of individuals; NoE: number of eggs; “Age of half-vulnerability” indicates the age at which 50% of the individuals are vulnerable to survey and commercial gears indexed by 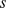 and 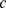, respectively.

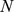	Cod abundance in January (NoI)	E1–E3, E7, D1–D2
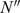	Cod abundance in August (NoI)	E14, D4–D5
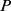	Cod predated by seals (NoI)	D1
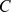	Cod commercial catch (NoI)	E16, D3, S4
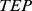	Total egg production (NoE)	E3, E13
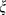	Sex ratio	E3
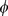	Proportion of maturing females	E2–E3
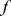	Fecundity (NoE 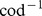 )	E3
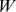	Cod weight (t)	E1, E4–E5
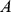	Last-age group	E1–E4, E14–E17
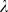	Functional responses of harp seals to cod	E7–E8
	(nb. consumed 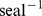 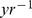 )	
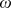	Age proportions of cods eaten by seals	E4
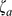	Baseline attack rate for age 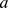	E7
	(nb. attacks 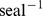 )	
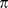	Normalization coefficient of attack rates	E7
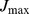	Maximum cod consumption rate	E6–E7
	(nb. consumed 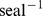 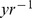 )	
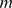	Shape parameter of the Holling response type	E7
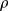	Feeding time spent by seals in Gulf each year (d)	E6, E8
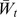	Cod mean weight for age groups targeted by seals (t)	E4–E5
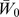	Cod mean weight for age groups targeted by seals	E8
	from 1998 to 2001 (t)	
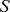	Seal abundance (NoI)	E6,E8
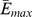	Maximal biomass of cod consumed by seal (t)	E5
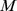	Residual natural mortality rate of cod ( 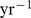 )	E9
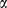	Intercept of the 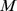 curve ( 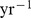 )	E9
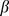	Slope of the 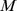 curve	E9
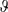	Asymptote of the 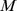 curve ( 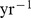 )	E9
	(residual mortality at last ages)	
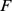	Fishing mortality rate of cod ( 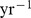 )	E11–E12
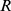	Cod recruitment at age 0 (NoI)	D5
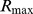	Maximum nb. of cod recruits (NoI)	E13
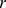	TEP needed to produce recruitment 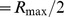 (NoE)	E13
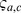	Commercial selectivity-at-age	E11
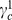	Shape parameter of the commercial selectivity	E11
	(1984–1993)	
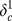	Age of half-vulnerability (1984–1993)	E11
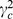	Shape parameter of the commercial selectivity	E11
	(1994–2006)	
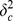	Age of half-vulnerability (1994–2006)	E11
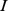	Abundance index (NoI)	E14–15,S1
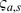	Survey selectivity-at-age	E14
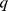	Survey catchability	E14
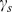	Shape parameter of the survey selectivity	E14
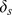	Age of half-vulnerability	E14
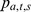	Proportion of number-at-age in the survey	E15, S2
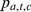	Proportion of commercial catch-at-age	E17, S3

### SIMCAB Estimation Model

SIMCAB is a deterministic age-structured model of the NGSL cod stock forced by harp seal predation and the environment through fish condition [43]. The model notations, relationships between state variables and parameters, and deterministic process equations are given in Tables 1–3. The model covers the time period from the year 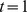 to 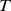 and the cod age classes from age 1 to 13. The fate of abundance 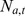, at the beginning of year 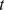 and for cods of age 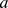, is as follows. Cods are firstly predated by seals, that remove a fraction 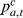 (Equation (D1) in Table 3) of 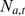. Defined as a predation probability, this fraction is defined by the choice of a Holling [50] functional response specific to generalist predators (Equation (E6–8) in Table 2), depending on the abundance 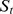 of seals. Then the removals due to commercial fishing occur at middle year (Equation (D3–4)). Between both processes a proportion of the fish population dies from other natural (residual) causes (Equation (D2) and (D5)), explained by environmental conditions. More precisely, the residual mortality rate 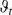 at the maximum age of cod is a function of cod condition 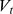 (Equation (2) in File SIM), which is itself dependent on water temperature. More details about these relations are provided in the next section, that describes the stochasticity involved within the projection model.

**Table 2:** Process components, observation functions and associated equations in the SIMCAB model. Notations 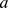, 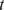, 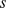, 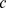 index age, year, survey, and commercial, respectively. The term 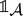 is the indicator function of event 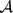.

	State moments	
(E1)	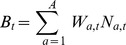	Biomass
(E2)	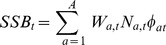	Spawning stock biomass
(E3)	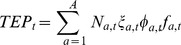	Total egg production
	**Mortality components**	
(E4)	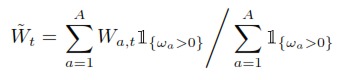	Mean mass of attacked cod
(E5)	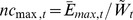	Max. consumed number
(E6)	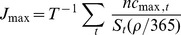	Mean max. consumpt. rate
(E7)	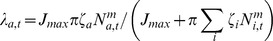	Functional response
(E8)	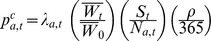	Predation probability
(E9)	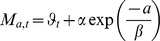	Residual mortality rate
(E10)	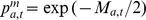	Residual half-death probability
(E11)	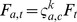 with 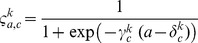	Fishing mortality rate
(E12)	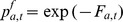	Fishing death probability
	**Recruitment component**	
(E13)	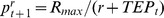	Egg hatching probability
	**Observation functions**	
(E14)	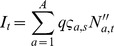 with 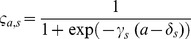	Survey indices
(E15)	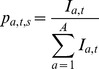	Survey-at-age obs. probability
(E16)	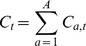	Total catch
(E17)	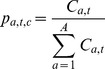	Catch-at-age obs. probability

**Table 3:** Deterministic and stochastic processes used in SIMCAB estimation and projection models, respectively. iid: independent and identically distributed; 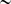: distributed as. 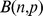: binomial distribution with probability parameter 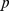.

Internal deterministic processes: estimation		
(D1)	Cod predation	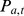 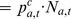
(D2)	Residual mortality 1	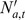 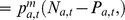
(D3)	Commercial fishing	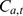 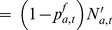
(D4)	Middle-year abundance	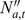 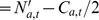
(D5)	Residual mortality 2	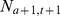 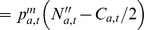
(D6)	Recruitment at age 0	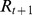 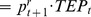
(D7)	Recruitment at age 1	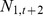 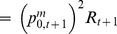

Two structural modifications were made with respect to the original choices made in [43]. Firstly, the mean mass of cod predated by seals was weighted by the proportions-at-age of cod eaten by harp seals estimated from a statistical model linking age classes and cod otoliths found in seal stomachs between 1998 and 2001 [43]. Secondly, the recruitment at age 1 in year 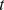 was derived from the total egg production (TEP) in year 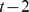 following a [51] relationship to account for the time delay in fish recruitment.

The full abundance matrix can be assessed by estimating the unknown parameter vector 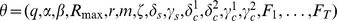 (Table 1). Maximum likelihood estimation of 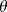 was enabled by the knowledge of noisy observations of survey abundance indices (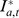), proportions of catch-at-age in the survey (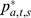) and commercial data (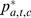), and total catches (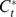). Abundance indices available from summer surveys were associated with cod abundances predicted in August, i.e. in the middle of the fishing season (Equation E14, D4, and S1). Observation equations linking the processes to the data are given in Table 4. Lognormal distributions including the Laurent correction [52] were used for the likelihood of catch (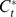) and relative abundance (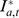) data. Furthermore, the likelihood of relative abundance data accounts for age-dependent effects between observations [53].

**Table 4:** Observation equations for the SIMCAB estimation model. iid: independent and identically distributed; 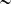: distributed as; 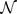: normal distribution; *Dir*: Dirichlet distribution.

(S1)	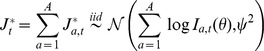	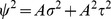
where	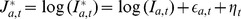	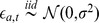
		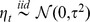
(S2)	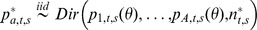	
(S3)	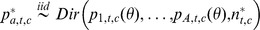	
(S4)	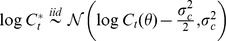	

True total catches 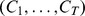 were constrained not to be lower than observed landings during 1984–1992, namely considered as right-censored data in the likelihood [54]. After the moratorium of 1993, fishing was more severely controlled and true catches after 1993 were assumed to stay between the observed landings and at most 110% of these values (namely, a only small positive bias, for instance due to discards of small fish, was allowed to occur during this period). The same approach was used to account for missing values among survey-at-age indices by considering total log-indices of concerned years as right-censored data. Multinomial distributions were chosen for catch-at-age proportions. Series of Nelder-Mead algorithms [55] were run with various starting points, then refined by simulated annealing techniques, and led to stable maximum likelihood estimation (Table 1 in File SIM).

### Projection Model

Model projections included both environmental stochasticity in cod recruitment and mortality components and uncertainty in parameters that were considered of primary importance to cod population dynamics. First, process errors (including environmental noise, uncertainties due to human activities…) were included in the SIMCAB propagation model through the use of binomial distributions for harp seal predation, residual mortality, commercial catch, and recruitments at age 0 and 1 (Equation P1–P6 in Table 3). Binomial distributions with random probability parameters are appropriate for modelling stochasticity in age-structured population models [56], [57] since the resulting variability (i) is unbiased and bounded (ii) is transmitted from the whole population to any subsample of the population, and (iii) increases at low abundance consistently with patterns observed in fish populations [58] (cf. File SIM). Furthermore, the statistical features of this noise do not need to be estimated (as it would be required for instance for classic lognormal variances). Second, variability was included in the model projections to describe the major sources of uncertainty in the SIMCAB parameters, which were selected from pre-experimental numerical tests. Stochasticity was introduced in the initial cod abundance, the relationship linking the CIL temperature anomalies and cod condition, the proportions of condition-at-age, and the weights-at-age (Table 5). A coefficient of variation of 20% for cod numbers was considered in the year before projection (i.e., 2009) to reflect the uncertainty in abundance estimates in the SIMCAB model. This was empirically estimated from a bootstrap distribution of the estimation error of this quantity, arising from the estimation model, build from the result of 30 simulated annealing algorithms with randomized initializations. The level of noise in the CIL temperature-condition relationship was inferred from the variability in the residuals of a linear regression model fitted to a dataset of CIL temperature anomalies [59], [60] and spring cod condition from 1984–2009 (Equation (1) and Figure 11 in File SIM). The resulting stochasticity would then propagate to the total egg production through fecundity modelled as a function of length and condition [20] and to the natural mortality. Indeed, the yearly residual natural mortality rates for old cod (asymptote 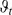) were related to condition through a decreasing linear function (cf. Equation (2) in File SIM) based on the results of laboratory experiments relating natural mortality to cod condition [21]. Proportions of condition-at-age were assumed to follow a Dirichlet distribution of parameters ***μ_α_*** estimated by maximum likelihood [61] using data over the period 1997–2009 (Table 5). Finally, random variations in the prospective yearly values of weight-at-age 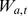 were included in the projections through the parameter 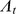 of the allometric length-weight relationship (cf. Equation (5) and Figure 12 in File SIM). No uncertainty was included in the sex ratio (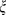), maturity (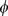), and length-at-age that were not considered major drivers of cod biomass projections relative to other parameters such as initial abundance and recruitment. These parameters were found to vary little over 1984–2009, i.e. CV 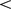 10% over the ages for 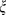 and 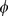 and CV close to 1.5% for length-at-age over the ages, and their values were set to the average for the period 2002–2009 for the projections.

**Table 5:** Stochasticity in the input parameters for the SIMCAB projection model. The notation 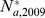 indicates the estimated abundance at age 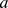 in 2009 from data between 1984 and 2009.

(N1)	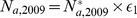	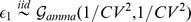
(N2)	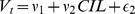	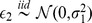
(N3)	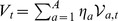	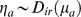
(N4)	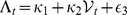	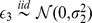

### Projection Scenarios

Projection scenarios were conducted considering changes in seal predation, environmental conditions, and fishing through the seal abundance (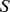), the cold intermediate layer (CIL) temperatures, and alternative harvest control rules (HCRs), respectively (Table 6).

**Table 6:** Projection scenarios considered in the analysis. CIL = cold intermediate layer. In each case, three fishing strategies are considered (from current practice to moratorium).

CIL anomaly (°C)		Seal mean abundance *(number of individuals)*
0.25 *(current water condition)*	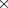	1.949. 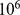 *(no reduction)*
−0.5 *(water cooling)*		1.364. 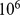 *(30% reduction)*
0.75 *(water warming)*		0.974. 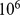 *(50% reduction)*

Changes in seal abundance would have a direct impact on cod removals through the multi-age functional response (Equation E4–E8 in Table 2). Three levels of seal abundance were considered for the projections over 2010–2040: (i) no reduction, i.e. the harp seal abundance was set to the abundance averaged over 2002–2009 [38], and reductions in 30% and 50% of the maximum abundance corresponding to the reduced catch reference points identified for the harp seal population of the Northwest Atlantic [44].

**Figure 2:**
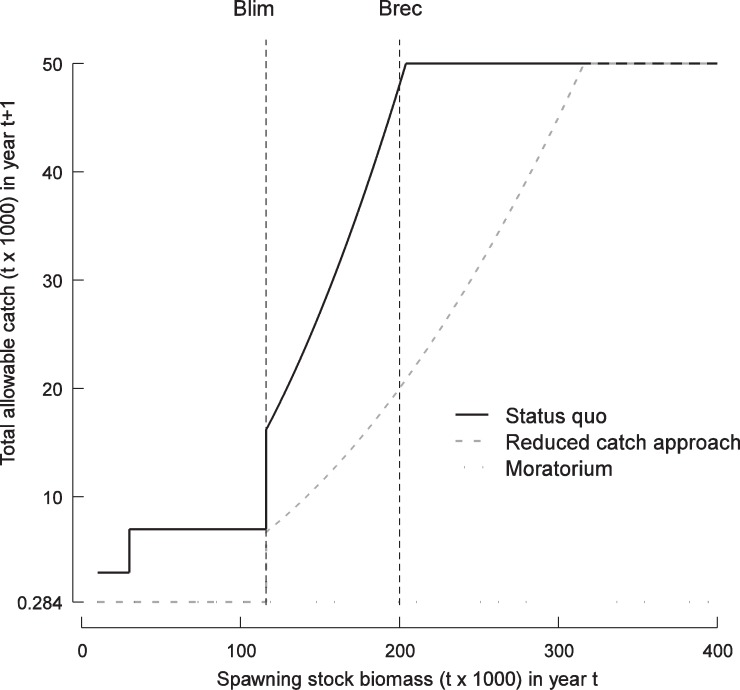
Linear regression of cod condition 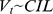. The adjusted Pearson's coefficient 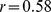, with 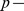
value 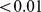.

Temperature is an essential environmental factor that does not only directly affect fish population dynamics, i.e. survival, growth, feeding rates, and movements, but also acts as a useful proxy for other physical and oceanographic processes regulating their prey and predator distribution, as well as metabolic kinetics in aquatic food webs [62], [63]. The CIL is characterized by strong inter-annual changes in temperature (cf. Figure 9 in File SIM) that have been shown to affect the early life stages of cod in the NGSL [64], [65]. In addition, the low water temperatures of the NGSL observed during the mid-1990 s have been hypothesised to negatively affect cod reproduction, growth, condition, and natural mortality [21], [46], [66]. The mean annual temperature of the GSL was therefore used as an indicator of environmental conditions in the NGSL. Following an approach proposed by [20], we established a linear relationship between cod condition in year 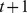 and the GSL temperature at 250 m deep in year 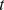 so as to link the environment to cod biology in our model projections (Figure 2). Water temperatures were derived from a large dataset of temperature profiles collected in the GSL and expressed as anomalies, i.e. deviations from their long-term mean calculated for the 1971–2000 reference period [67] (Figure 10 in File SIM). The fit was made using an usual least square method. Predicting the NGSL environmental conditions over the next decades remains however challenging regarding the peculiarities of the Gulf topography and water circulation, and the complex intricate effects that climate change could have on sea ice extent, light attenuation, freshwater runoff, and nutrient supply which all drive ecosystem productivity [68]–[70]. To cover a large range of environmental conditions in the Gulf of St. Lawrence, three temperature regimes based on the historical time series of the CIL during 1984–2009 [60] were simulated to represent mean and extreme scenarios, as usual in sensitivity analysis [71]. The CIL temperature anomaly averaged over 1984–2009 was considered as the base case scenario while the temperature averaged over the 5 hottest and coldest years observed in the time series were considered for the warming and cooling scenarios, respectively.

**Figure 3:**
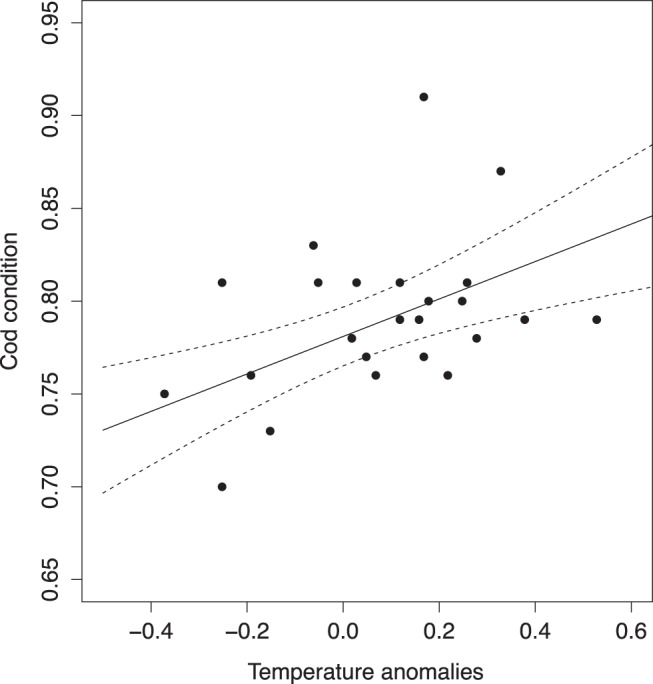
Harvest control rules (HCRs). They determine the total allowable catch (TAC) of the NGSL cod stock in year t+1 from the spawning stock biomass (SSB) in year t. B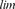 and B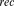 are the limit and recovery biological reference points, respectively.

Three HCRs characterized by increasing levels of conservation were considered to represent alternative ways of managing the cod stock (Figure 3). Here, the HCR determines the total allowable catch (TAC) in year t+1 from the knowledge of spawning stock biomass (SSB) in year t. A status quo HCR was derived from the past values of SSB estimated from sequential population analysis and subsequent allocation of TACs [18]. A reduced catch HCR was also used for the projections, leading to lower TACs than the status quo HCR for a given level of SSB (Figure 3). Finally, an HCR based on a moratorium was used in the projections to simulate the closure of the cod fishery. In these scenarios, the TAC was set to 284 t, which corresponded to the mean annual catch observed during the 1994–1996 moratorium and due to bycatch in other fisheries than the cod fishery. Although the moratorium differs from the adaptive reduced catch approach, it results in quite similar levels of catch removals for low cod abundance, i.e. when spawning stock biomass 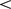 116,000 t (Figure 3).

Projection results were investigated through the stochastic spawning stock biomass trajectories over 2010–2040, the time periods required for the median SSB to reach extirpation, two biomass reference points B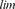 and B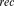 that are traditionaly usedin reduced catch managements, and the probability of exceeding B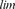 and B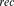 at each time step. B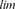 is defined as the limit of recruitment overfishing, below which the stock is considered to have suffered serious or irreversible harm. B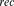 is the recovery biological point above B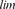, below which the stock's productivity can be considered suboptimal. In this study, B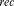 was set to 200,000 t, according to the conclusions of a Zonal Assessment Meeting in 2003 [72]. This value corresponds to a healthy stock state since it has been recently slightly rescaled to 180,000 t to account for newly detected changes in weights at age [73]. The more critical value of B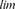 having recently been reevaluated from 90,000 t [72] to 116,000 t [73], this latter value was considered in the present study. Along the paper, the terms “partial recovery” or simply “recovery” will refer to the reaching of B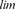, while “complete recovery” will refer to exceeding B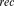.

## Results

### Model Testing

The model validation procedure coupled with the retrospective analysis indicated that observations predicted from the model were consistent with the annual survey indices observed during 2006–2009. For all years and projection models, the 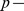
values relating survey indices and catches with their corresponding predictive distributions were found between 0.319 and 0.584 (Tables 7). These p-values can be understood as the tail orders of the observed indices with respect to their simulated distribution using the model. The idealistic best fit, namely the perfect accordance of the model and the observations, should provide a tail order of 50%. Both simulated and observed indexes 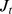 and 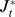, involved in this test, were summed over ages 2 to 11 only to account for missing observations at age 1, 12 and 13. These high values indicate that the observations of year 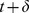 are in accordance with the projection model calibrated over years 1 to 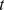, when 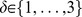 and 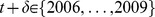. In File SIM are given (Table 6 in File SIM) the same 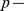
values defined for proportions-at-age for both survey and catches. Reaching magnitudes closer to 50%, they show an even better accordance of the simulation model with theobserved data. Over these years, this result make plausible the structural choices made for the model and legitimize its use for the technical exercise of projecting abundance from year 2009.

**Table 7:** 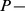
 values of survey indexes (summed over ages 2 to 11) and total catches (summed over ages 5 to 13) with respect to their respective predicted (propagated) distribution, calibrated by maximum likelihood estimation over the previous years.

Years of projection	Years of estimation			
	1984–2005	1984–2006	1984–2007	1984–2008
	Survey indexes			
2006	0.319			
2007	0.465	0.322		
2008	0.417	0.348	0.497	
2009	0.387	0.528	0.584	0.477
	Total catches			
2006	0.401			
2007	0.397	0.438		
2008	0.478	0.358	0.476	
2009	0.499	0.513	0.525	0.621

### Factors Explaining the Stock Recovery

**Figure 4:**
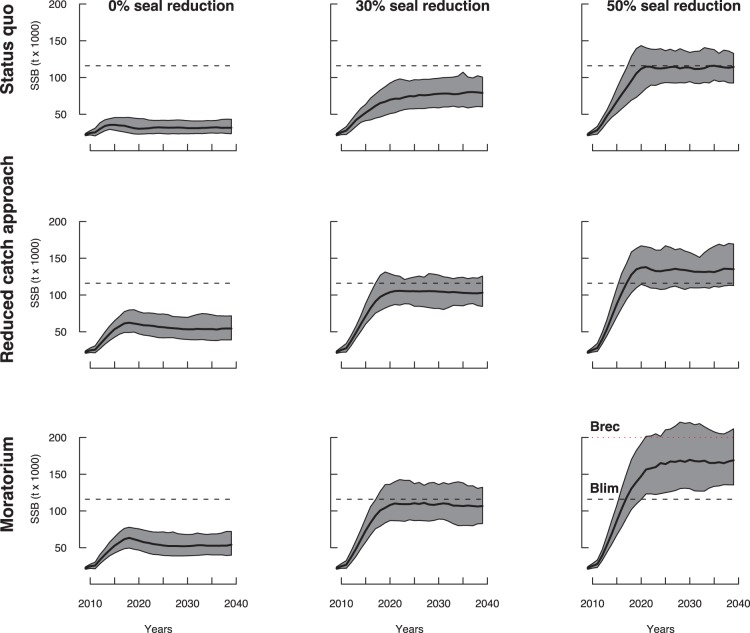
Cod SSB forecast in years 2010–2040 under water standard conditions (cold intermediate layer (CIL) anomaly  = 0.25°C). Plain lines and grey areas indicate median values and 90%-confidence domains, respectively. The dashed line indicate the limit of recruitment overfishing 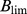. The red dotted line indicates the complete recovery point B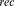.

**Figure 5:**
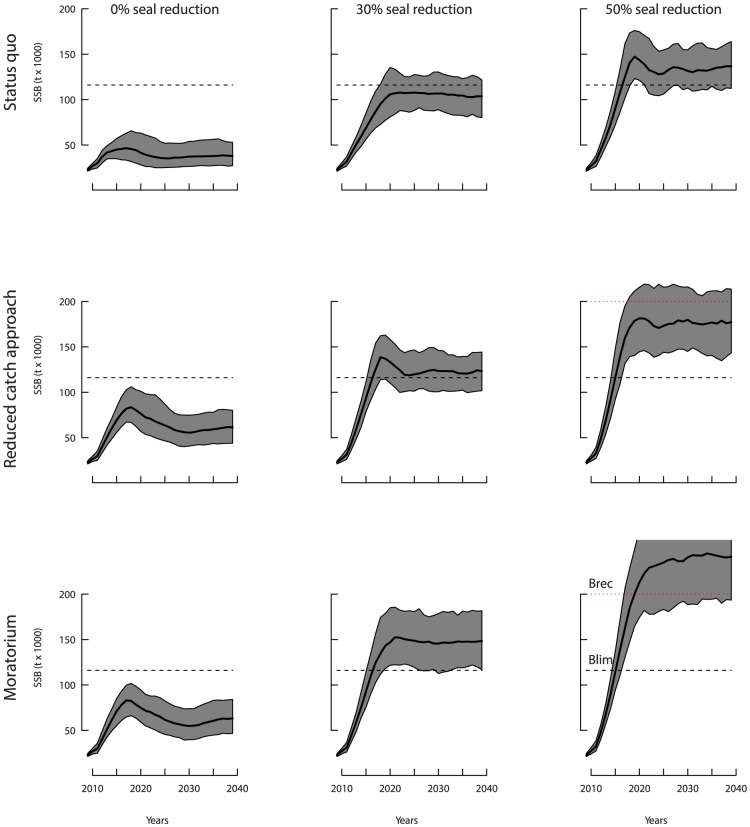
Cod SSB forecast in years 2010–2040 under water warming conditions (cold intermediate layer (CIL) anomaly  = 0.75°C). Plain lines and grey areas indicate median values and 90%-confidence domains, respectively. The dashed line indicate the limit of recruitment overfishing 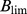. The red dotted line indicates the complete recovery point B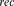.

**Figure 6:**
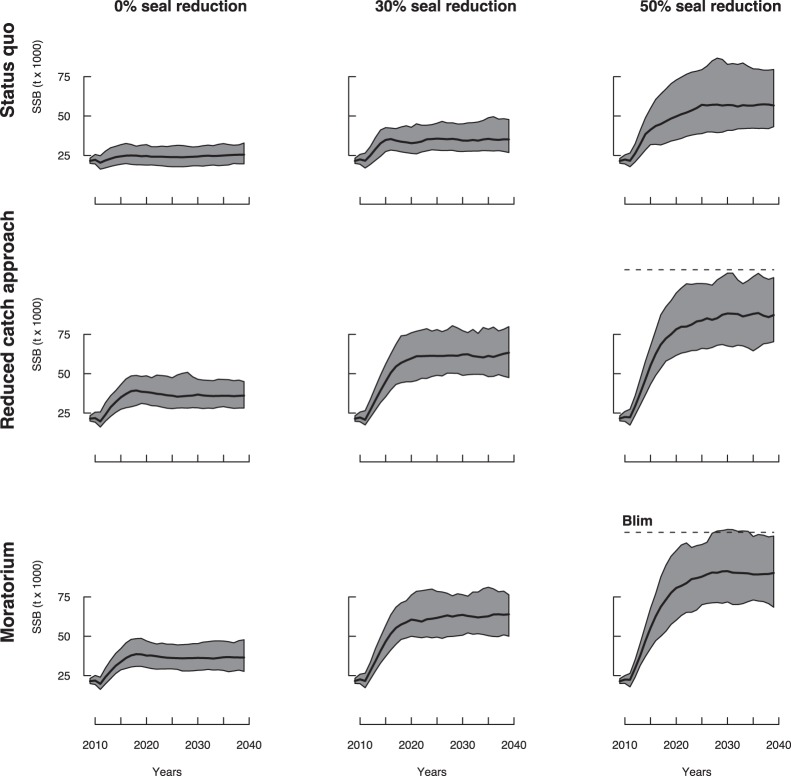
Cod SSB forecast in years 2010–2040 under water cooling conditions (cold intermediate layer (CIL) anomaly = −0.5°C). Plain lines and grey areas indicate median values and 90%-confidence domains, respectively. The dashed line indicate the limit of recruitment overfishing 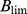.

In order of importance, water temperature, predation by seals, and harvest control rules affected the SSB trajectories projected into the future. The main factor affecting cod recovery was environmental; changes in the CIL temperature resulted in strong differences in the magnitude of spawning stock biomass changes and time span for recovery (Figure 4–6). Under current water temperature condition, i.e. CIL anomaly  = 0.25°, the current approach of managing the cod stock would permit stable partial recovery within the next 20 years only by reducing the seal population by 50% (Figure 4). Adopting more conservative management approaches, which would involve a 50% reduction in cod catch or a moratorium, the probability that the SSB attains B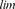 during the same period would reach a non-negligible value only if, in parallel, there was a substantial reduction in seal abundance, i.e. at least 30%. Such reductions would result in an increase in the SSB by 65% compared to the no reduction scenario, but the chances of cod reaching B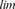 within the projection period would remain less than 50%, except if the seal population was reduced by 50%. In this most favourable case, however, the chances of reaching the complete recovery point B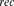 would only be 15% after 2030 (Figure 4).

Water warming conditions improved the speed of recovery, with chances of reaching B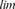 exceeding 50% after 2020 only if the seal population is reduced (Figure 5). If the harp seal population is reduced by 50%, there is an estimated 20% chance of a complete recovery above B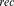 by 2025 if fishing is also reduced following an adaptive strategy. This probability increased to 87% if a moratorium was implemented.

Maintaining current management practices under cool water conditions (i.e., CIL anomaly = −0.5°) would maintain the population at low levels of biomass, close to below the current level of 20,000 t, regardless of the harp seal abundance in the ecosystem (Figure 6). Adopting a reduced catch or moratorium approach under similar conditions would only result in a slight increase in SSB, that would not reach values close to B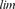 even under a 30% reduction in seal population. In the most favorable case considered (moratorium and 50% seal reduction), the chance of reaching B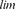 within the next 30 years would not exceed 6.5%.

There were generally few differences between the reduced catch and moratorium approaches at low levels of cod SSB since such levels would result in a closure of the cod fishery under the reduced catch management rule (Figure 3). This occurred for the scenarios of no seal reduction and water cooling. In other cases, a slight but clear difference in recovery time was evident between situations of a reduced catch or a complete moratorium.

### Time Span for Partial Recovery

**Figure 7:**
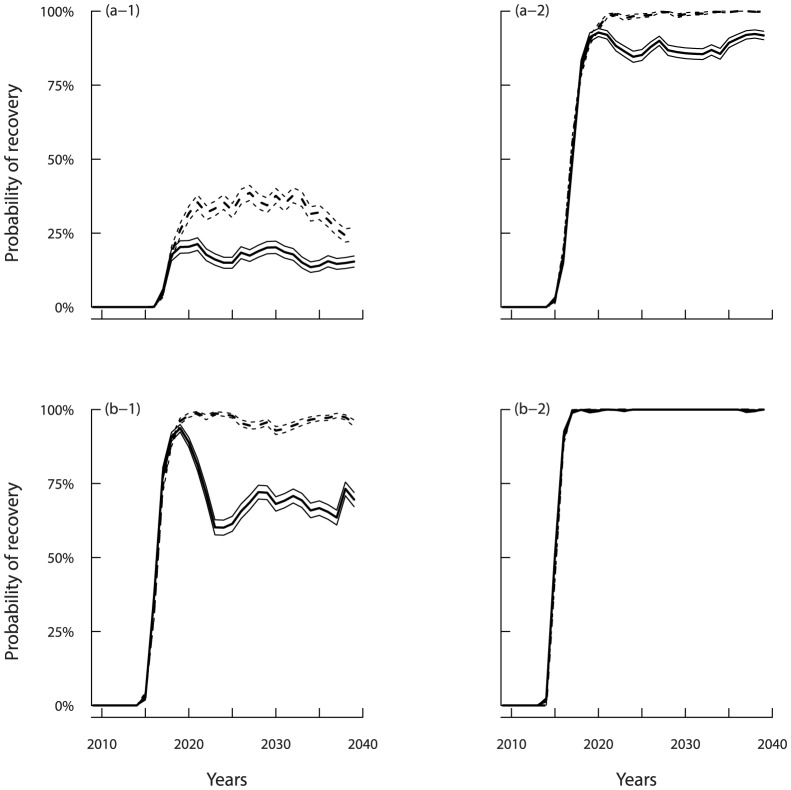
Probabilities-at-year for the SSB to exceed the limit of recruitment overfishing 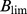. Alternative fishing management strategies, levels of seal reduction, and environmental conditions are considered. **(a)** Current environmental conditions (CIL = 0.25°C) for 30% (a–1) and 50% (a–2) seal reduction. **(b)** Warming environmental conditions (CIL = 0.75°C) for 30% (b–2) and 50% (b–3) seal reduction. Plain and dashed lines indicate the effects of reduced catch and cod fishery moratorium, respectively. Mean and 95% confidence intervals of the probabilities are displayed.

The time span required for cod recovery was strongly dependent on water temperature conditions. Applying more conservative management approaches and further reducing the seal population favoured more rapid recovery (Figure 7). For all scenarios leading to partial stock recovery, the reduced catch management rule and moratorium did not yield any difference in recovery times before 2020 as shown by the mutual coverage of the confidence intervals around the probabilities of recovery (Figure 7). After 2020, a statistically-significant separation appeared between the confidence intervals. The moratorium approach reduced the time for recovery, while the reopening of fishing through the reduced catch approach would have the effect of reducing the SSB during the following 10 years.

Under current water temperature conditions, cod would only recover within the time span considered provided that conservative management approaches would be implemented, coupled with a reduction in seal abundance by 30% or more (Figure 4). In this case, the beginning of partial recovery was only observed starting in 2018. The overall probability of recovery increased with time but remained below 40% until 2040 when the seal reduction was 30% (Figure 7a–1). For both seal reduction scenarios, the moratorium approach increased the median probability of recovery on average by about 10% starting in 2020 compared to the reduced catch approach.

**Figure 8:**
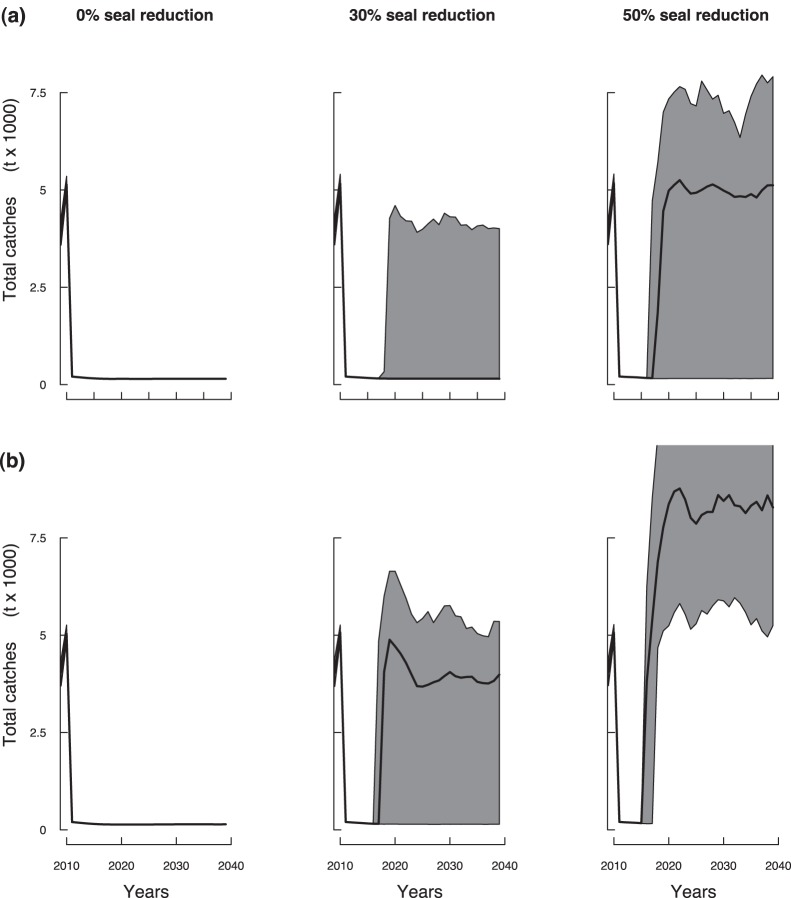
Cod total catches forecast in years 2010–2040 under water standard conditions. [**(a)**: CIL = 0.25°C] and warming conditions [**(b)**: CIL = 0.75°C], for the reduced catch fishing regime. Plain lines and grey areas indicate median values and 90%-confidence domains, respectively.

Under warming conditions, and a reduction of seal abundance by 30%, both moratorium and reduced catch approaches led to a similar fast partial recovery by 2017 (Figure 7b–1). However, under reduced fishing or a moratorium, a 50% reduction of seal abundance allowed the SSB to stabilize above the partial recovery point after 2020 (Figure 5). Under these water warming conditions, a reduced catch approach appeared to be compatible with reaching a recovery within another 10 years and an overall increasing fishery (Figure 8).

## Discussion

Our findings provide insights into how the combined effects of the physical environment and trophic interactions can affect the outcomes of fishing management strategies aimed at rebuilding depleted stocks. Modelling results indicate that the NGSL stock recovery will probably take a long time (at least 10 years) and is strongly dependent on water temperature conditions, followed by seal predation, and fishing strategies. According to model projections, current water temperature conditions and levels of fishing pressure are unlikely to result in the NGSL cod stock rebuilding within the next 30 years due to poor productivity. A significant reduction in fishing pressure would increase the levels of SSB, but this could only be achieved with a high probability if accompanied by large reductions in seal abundance. Under a warmer water temperature regime, conservative management approaches and reductions in the harp seal population would benefit the NGSL cod stock recovery by reducing rebuilding time. Projections also indicated that a decrease in water temperature, as observed during the stock collapse in the 1990 s and expected in the near future (as explained below) would likely result in the stock remaining below critical levels over the next 30 years.

Following our model, partial stock recovery might only be achieved under a significant reduction in seal abundance, provided the NGSL water temperature remains at current levels or warmer. Reductions in fishing will likely have an immediate effect on rebuilding, through protection of the SSB, whereas reductions in seal abundance will have a delayed impact, because seals primarily target small fish (10–15 cm), which will not be recruited into the SSB for several years. Fishing strategies based on reduced catch approaches could be prefered over a full moratorium as they could result in more socio-economic benefits. However, the cumulative effects of a moratorium approach and a 50% seal reduction seem to be needed to lead the stock towards the complete recovery by 2040. Some decline in harp seal abundance may occur naturally if poor ice conditions documented in recent years, which result in increased seal mortality, were to continue [37], [74].

### Modelling Population Dynamics at Low Abundance

Modelling population dynamics at low abundance remains a challenging task that can have strong implications in wildlife and fisheries management, as well as for conservation issues. A large database of several thousand time series of populations estimates for more than 1,000 species of birds, mammals, bony fishes, and insects revealed that the relationship between population growth rates and density is generally concave, i.e. most of populations would exhibit compensatory mechanisms at low abundance through density-dependent processes [75]. Based on a comparative analysis of 128 fish stocks, Myers et *al.* [10] showed evidence of increased survival rate at low abundance for the majority of the populations, which is consistent with compensatory mechanisms. The overall failure of fish populations to recover following collapses despite substantial reductions in fishing mortality however suggests that factors other than fishing can be considerably more important to recovery [7], [8]. Hence, depensatory mechanisms should not be dismissed for lack of recovery and factors such as predation, stock structure, and environmental influences also need to be considered in recovery analyses [11], [13], [76], [77]. Here, a compensatory stock-recruitment relationship [51] was used for the NGSL cod at age 0, while harp seal predation on young age classes was explicitly modelled to allow for the emergence of more complex recruitment processes.

Fishing is a size-selective process that has been shown to result in exploited species exhibiting higher temporal variability in abundance than unexploited species [51], [78]. At low stock size, recruitment variations propagate more through the population because of age-structure truncation that can affect both the reproduction patterns (e.g. spawning seasonality) and the reproductive potential (i.e. fecundity and egg and larva survival) of the stock [79], [80]. Fish populations at low abundance would then be mainly regulated by density-independent stochastic processes driven by extrinsic environmental forcing. The SIMCAB model incorporated some of these features through the effects of the Cold Intermediate Layer temperature on cod fecundity and natural mortality. Furthermore, a meta-analysis recently conducted on 150 fish populations concluded that there was increased survival variability during population declines resulting from density dependent processes [58]. Also, the natural heteroscedasticity in recruitment (i.e. non-constant variance) at low population levels should be considered in recovery projections because it may lead to higher extirpation risk [58]. The use of binomial distributions with random probability parameters in the SIMCAB model projections is consistent with such patterns by allowing for the coefficient of variation to increase when the population decreases (cf. File SIM).

### Prospective Research Avenues

The current status of the NGSL cod resembles that of numerous fish populations around the world, which calls for urgent management actions for rebuilding. Implementing recovery plans for depleted fish stocks however requires simple metrics for managers and stakeholders to monitor the stock status trends as well as adaptive policy management [81]. In absence of data produced from controlled experiments, modelling population dynamics can inform management decisions through the exploration of future scenarios [82]. Accounting for environmental interactions and uncertainties arising from model estimations through stochastic projections allows for such explorations. Then, as shown here, comparison procedures between projections and observed data are useful to test the possible irrelevance of the structural hypotheses behind the modelling.

Swain and Chouinard [83] performed projections for the cod population of the Southern Gulf of St Lawrence and concluded that the stock would continue to decline, even in the absence of fishing owing to high levels of natural mortality on adult cod. However, their biomass trajectories were based on a simpler model than the SIMCAB model and did not include any predation forcing nor environmental component. Recently, MacKenzie et *al.* [84] developed a similar modelling approach to conduct projections for the cod stock in the eastern Baltic Sea and assess the effects of seal predation mortality, salinity decrease, and exploitation on cod recovery. They also found that seal predation had a much lower impact on cod recovery than the effects of exploitation and salinity. Our stochastic approach can be seen as a generic and consistent statistical framework that can be applied to other depleted populations exposed to both fishing and predation pressures. Effort should be directed to increase the realism of the model by improving our knowledge of the sensitivity of model components to environmental variation, additional sources of predation mortality, and human decisions likely to affect the stock. Especially, in a perspective of improving the accuracy of projections and the management adaptiveness, the present sensitivity results highlight the need for a deeper modelling study of the environmental pressure and its links with prospective climatic scenarios.

Such scenarios predicted by the Intergovernemental Panel on Climate Change (IPCC) suggest that strong changes in the marine ecosystems of the northwest Atlantic could occur in the forthcoming years [85]. In particular, an accelerated sea-ice melting in the Arctic and changes in the Arctic Ocean circulation have already been observed since the early 1990 s and might result in freshwater exports into the Atlantic Ocean with strong consequences on marine food webs [86], [87]. The intrusion of Labrador Shelf colder waters into the Gulf of Saint Lawrence could lead to the cooling of the intermediate layer with major consequences not only on cod condition but on the major plankton communities and upper trophic levels of the Gulf of Saint Lawrence [88], [89].

In this study, we examined water temperature effects and those of a single predator. However, the NGSL is a much more complex ecosystem with extensive trophic linkages [41], [90]. Failure to consider these linkages in any management activity could lead to unexpected and counter intuitive effects [40], [91]. Among them, the possible negative feedback effects of the decline of the resource on seal population have not been investigated yet, although indications that the decline of reproductive success and health of seals could be correlated to a decline of fish stock were noticed in the past [92]. Understanding those mecanisms would result in transforming the SIMCAB model, which is focused on a single population submitted to exogeneous forcings, to a true predator-prey model. More globally, further exploration of the linkages between environmental changes and food web dynamics could be addressed with end-to-end ecosystem models [93]. However, these require large data sets as well as major assumptions on key factors driving the functioning of marine ecosystems and much work remains before they become operational.

Ideally, a projection model should take into account all sources of uncertainty inherent to (i) the model structure and (ii) the parameter estimation [94]. The first issue can be tackled by relaxing the deterministic features of the model and including environmental noise, i.e. process error in the structural equations, as implemented here. Addressing the issue of uncertainty in the model parameters requires having a precise idea of how all parameter estimators evolve within their confidence domain. In the present study, projections focused on main influences on cod recovery and included stochasticity in several key parameters since accounting for all sources of uncertainties in projections was found to be computationally intensive, and could reduce the readability of the results with regards to the major input parameters. A better grasp of modelled uncertainty could arise from systematic sensitivity analyses [71], [95]. For instance, the sensitivity of some model assumptions about the seal-cod predation process, particularly diet composition, could be tested.

However, such analyses remain difficult to conduct because of very intensive computational time requirements [96], and perhaps more important suffer from the absence of clear methodologies of global sensitivity analyses adapted to stochastic models or computer codes [97], [98], whose inputs mix stochastic variables (biological processes) and categorical processes (agent-based decisions and actions). Further work to include all sources of variability in the model through the development of a full Bayesian version of SIMCAB would provide a more structured framework to understand the impacts of uncertainties on projection results, and eventually improve management advice. Methodological developments are needed in the forthcoming years to address these topics that will chiefly benefit from the increasing development of parallel computing [99] and computer code emulation [100].

## Supporting Information

File SI Supporting Information for the article Forecasting the major influences of predation and environment on cod recovery in the northern Gulf of St. Lawrence.
(PDF)
