# Supplementary material for: Forecasting the Major Influences of Predation and Environment on Cod Recovery in the Northern Gulf of St. Lawrence
Source: PLoS One. 2014 Feb 11;9(2):e82836. doi: 10.1371/journal.pone.0082836 (PMC3921123; doi:10.1371/journal.pone.0082836)
Supplement: File SI — Supporting Information for the article Forecasting the major influences of predation and environment on cod recovery in the northern Gulf of St. Lawrence. (PDF) [file pone.0082836.s001.pdf]

Supporting Information for the article

Forecasting the major influences of predation and environment on cod recovery in the northern Gulf of St. Lawrence

Nicolas Bousquet[1], Emmanuel Chassot[2], Daniel E. Duplisea[3], and Mike O. Hammill[3]

[1]*EDF R&D, MRI Dpt, 6, quai Watier, 78 401 Chatou, France*

[2]*IRD CRH, Avenue Jean Monnet, BP 171, 34 203 Sète Cedex, France*

[3]*IML Fisheries and Oceans Canada, 850 route de la Mer, Mont-Joli, Québec G5H 3Z4*

Introduction

This document details the main technical aspects in the estimation and projection of the Seal IMpact on Cod Abundance (SIMCAB) model carried out in the aforementioned article (denoted BCDH in the following). First, we provide estimation results for the SIMCAB model on the period 1984-2009 and summarize a retrospective analysis testifying of global model robustness conditional to the knowledge of the seal abundance. We then give methodological arguments for the choice of binomial environmental stochastic mechanisms listed in Table BCDH.4. Finally we provide linear regression results that are needed to infer the level of noise in the Cold Intermediate Layer (CIL) temperature-condition relationships, which are used for the projections, and a last paragraph is dedicated to the computation of projective fishing rates in function of simulated Harvest Control Rules (HCR). Some figures and tables are finally inserted to complete the results provided in the article.

Supplement S1. Estimation results for the SIMCAB model on the period 1984-2009

The SIMCAB estimation model (cf. § 1 in Methods in BCDH) is a modified version of the original model described in Chassot et al. (2009). In a first study, both models were compared in estimation when fitted on the original 1984-2006 data used in Chassot et al. (2009). For this new model, the minimum value of the objective function (the negative log-likelihood) was 5,271, leading to an Aikake Information Criterion (AIC; Aikake 1974) of 9,481, which improved significantly the result obtained in Chassot et al. (2009) (AIC = 12,373). A similar improvement was noticed when both models were fitted on the seal and cod data described in BCDH: the AIC characterizing the first version of the SIMCAB model was 11,816 while the second version reached the value 8,906. Main estimated parameter values are provided in the summary Table S1 and the consistant estimation of theoretical survey indices, accounting for *censored* observations (missing values for certain ages), illustrates the capacity of SIMCAB to reproduce observable behaviors (Fig. S2).

The main differences between the two versions of SIMCAB appearing during the 80's correspond to a period when observations were surprising in regards of the suspected overfishing. Udpated total catches are displayed on Figure S3, showing the overfishing quantified by the censored terms in the likelihood

(especially before the 90's). Especially, it was found that both landings and first MLE estimates are not always covered by the usual 95% confidence area produced by the assessment method for the first years (before 1994). This overfishing was besides reflected by slightly higher fishing rates than those estimated by the original version of SIMCAB (Chassot et al. 2009). A delayed stock-recruitment relationship, in parallel of accounting for missing data in survey and catches, was found to improve the flexibility of SIMCAB. This is traduced by the proximity of the estimated SSB to the SSB estimated by a basic sequential population model (Fig. S4) extremely governed by the data.

Supplement S2. Retrospective study

A retrospective cohort study allows to test the robustness of the model by checking the occurence of systematic patterns of bias in the successive estimates of the main quantities involved [4]. In this aim, SIMCAB was first estimated on the 1984-2005 data, then reestimated by adding additional years of data until reaching 2009. The estimation results of the main unidimensional parameters are provided on Table S2. Most of estimates appeared stable over last years. The estimations of fishing and exploitation rates (Fig. S5 and S6) showed similar consistant patterns, especially after the year 2000, which are in accordance with those estimated from 1995 by virtual population analysis [5, 6]. The wide fluctuations of the estimates noticed before 1990 have however a limited effect on the stability of the estimation of the SSB, which appears robust throughout this analysis (Fig. S7). When fitted over the years 1984-2005 and 1984-2006, the part of natural mortality due to seal predation was estimated lower than when accounting for more recent data. This variation is correlated with the significative increasing of the seal population from 2006 with respect to it mean level in 2002-2005 [7]. The observed negative correlation between the parameters impacting seal predation (ξ, π) and residual mortality (α, β) implied that the stock-recruitment relationship was estimated little productive in 2005 and 2006 (Fig. S7). In a coherent way with the increasing of the seal predation, impacting small cods only, the relationship between the spawning stock and the recruits reaches upper levels when data from 2007-2009 are took into account. An overview of total mortality results (Fig. S8) does not indicate strong temporal patterns similarly to [1], but the increase of the natural mortality rate over the last years fits is consistent with a similar phenomenon affecting the CIL temperature [8] and the assessments chosen for simpler models [9].

Supplement S3. Methodological arguments for eliciting binomial environnemental noises in structural equations

Urn population hypothesis

Inserting process error inside population equations requires assessing one or several measures of variability (typically, standard deviations of lognormal distributions, cf. Haddon 2001). A urn hypothesis about the behavior of the cod population can tackle this issue. Basically, the ecosystem is assimilated to a urn where balls with different colors represent cod categories inside a same yearly population: fish that will die from natural cause or fishing, or survive. Extracted populations are thus described as random sums of independent Bernoulli removals from the urn, and also follow binomial distributions conditionally to the size of the entire population. This assimilation remains clearly crude (fish in a given age class are perceived as independent entities) but the induced stochastic population processes are unbiased and theoretically bounded (unlike lognormal distributions). It has the supplementary advantage not to introduce nuisance parameters in state-space models under the form of variances of environmental noises, which reduces the risks of overparametrization.

Such urn models are used in a large number of applications, especially in population genetics (Donelly

Table S1. Parameters fixed or estimated in SIMCAB. NoI: number of individuals. NoE: number of eggs. NS: data not shown. Estim.: estimated. Calc.: calculated. “Age of half-vulnerability” indicates the age at which 50% of the individuals are vulnerable to fishing or survey gear. Units are same as those in Table.BCDH 2. The symbols e+/e- are for the positive and negative exponential functions.

Notation	Definition	Type	Value or Origin
ξ	Sex ratio	Fixed	[3]
ϕ	Proportion of maturing females	Fixed	*ibid.*
f	Fecundity (NoE cod^{-1})	Fixed	*ibid.*
W	Cod weight (t)	Fixed	*ibid.*
$\boldsymbol{\omega}$	Age proportions of cods eaten by seals	Fixed	*ibid.*
ζ_a	Baseline attack rate for age a	Fixed	*ibid.*
π	Normalization coefficient of attack rates	Estim.	0.11
$J_{\max}$	Maximum cod consumption rate	Calc.	1793
m	Shape parameter of the Holling response type	Estim.	1.98
ρ	Feeding time spent by seals in Gulf each year (d)	Fixed	150
$\bar{W}_t$	Cod mean weight for age groups targeted by seals (t)	Fixed	*ibid.*
$\bar{W}_0$	Cod mean weight for age groups targeted by seals from 1998 to 2001 (t)	Fixed	288e-6
S	Seal abundance (NoI)	Fixed	*ibid.*
σ	Total catches standard deviation	Estim.	0.39
ψ	Log total survey standard deviation	Estim.	9.80
α	Intercept of the M curve (yr^{-1})	Estim.	5.04
β	Slope of the M curve	Estim.	0.90
ϑ	Asymptote of the M curve (yr^{-1})	Calc.	*ibid.*
$R_{\max}$	Maximum nb. of cod recruits (NoI)	Estim.	e+23.51
r	TEP needed to produce recruitment = $R_{\max}/2$ (NoE)	Estim.	e+28.39
γ_c^1	Shape parameter of the commercial selectivity (1984-1993)	Estim.	1.73
δ_c^1	Age of half-vulnerability (1984-1993)	Estim.	7.85
γ_c^2	Shape parameter of the commercial selectivity (1994-2009)	Estim.	2.66
δ_c^2	Age of half-vulnerability (1994-2009)	Estim.	5.40
q	Survey catchability	Estim.	5.32
γ_s	Shape parameter of the survey selectivity	Estim.	2.99
δ_s	Age of half-vulnerability	Estim.	2.72

Table S2. Retrospective estimations of main unidimensional parameters. The parameter ξ is the total attack rate (i.e., the sum of calibrated attack rates $\pi\xi_a$ over ages)

	**1984-2005**	**1984-2006**	**1984-2007**	**1984-2008**	**1984-2009**
$-\ln\xi$	11.85	12.08	12.95	12.99	12.94
π	0.05	0.07	0.11	0.10	0.11
γ_s	3.36	3.17	3.00	2.99	2.99
δ_s	2.22	2.27	2.58	2.64	2.72
γ_c^1	1.69	2.02	1.65	1.64	1.73
δ_c^1	5.89	5.00	7.71	7.61	7.85
γ_c^2	2.00	2.12	1.96	1.96	2.66
δ_c^2	5.88	5.64	5.40	5.40	5.40
m	2.20	2.10	1.99	1.96	1.98
α	5.45	5.30	5.05	5.06	5.04
β	0.92	0.92	0.90	0.90	0.90
$\ln R_{\max}$	23.28	23.57	23.54	23.46	23.51
$\ln r$	30.08	30.60	29.65	28.55	28.39

and Kurtz 1996) . Close models have been adapted to population of birds by Dupuis (2002). Virtala et al. (1998) have used this approach to define a general VPA-based model with process error. Their catches were however supposed to be known, which induced behaviors close to determinism; in the projection study conducted in BCDH they are sampled as random variables, which allows for the advantages listed hereinafter. Following similar ideas in a Bayesian framework to define the population dynamics of cod along the Norwegian Coast, Follestad (2003) elicited hierarchical priors that are especially based on the assimilation of survival processes with Bernoulli trials.

Propagation of uncertainties

Essentially two benefits are due to the binomial mechanism of population decreasing. First, it appears that a high population relative variability is nearly fully transmitted to a subsample of this population. Indeed, assume that discrete random variables N and N' are linked by

$$N' \quad \sim \quad \mathcal{B}(N,p)$$

where p is a probability. Then $\mathrm{E}[N'] = p\mathrm{E}[N]$ and $V[N'] = \mathrm{E}[N'^2] - p^2\mathrm{E}^2[N]$. Thus

$$\mathrm{CV}[N'] \quad = \quad \sqrt{\frac{\mathrm{E}[N'^2]}{p^2\mathrm{E}^2[N]} - 1}.$$

Since $\mathrm{E}[N'^2] = \mathrm{E}\left[\mathrm{E}[N'^2|N]\right] = \mathrm{E}\left[p^2N^2 + p(1-p)N\right]$, then

$$\begin{aligned}\mathrm{CV}[N'] &= \sqrt{\frac{\mathrm{E}[N^2] + (1-p)\mathrm{E}[N]/p}{\mathrm{E}^2[N]} - 1},\\ &= \sqrt{\mathrm{CV}^2[N] + \frac{E^2[N]}{E^2[N]} + \frac{(1-p)}{p\mathrm{E}[N]} - 1},\\ &= \mathrm{CV}[N]\sqrt{1 + \frac{(1-p)}{p\mathrm{E}[N]\mathrm{CV}^2[N]}},\end{aligned}$$

hence, when the CV of N increases, $\mathrm{CV}[N'] \simeq \mathrm{CV}[N]$.

The second benefit is that it implies higher demographic stochasticity when the population abundance decreases at low levels, consistently with patterns observed in fish population [14]. Indeed

$$\mathrm{CV}[N'] = \sqrt{(1-p)}\sqrt{\mathrm{E}[N']}^{-1}$$

which increases when $\mathrm{E}[N']$ decreases.

Supplement S4. Inference of environmental noise in CIL-dependent stochastic processes

We focus here on the inference on regression parameters which link the CIL value to the cod condition (Equ. BCDH.N2-N3) and the latter to the cod length (Equ. BCDH.N4).

Values for the parameters of the linear regression

$$\mathcal{V}_t = \nu_1 + \nu_2\mathrm{CIL} + \epsilon_1 \quad \text{with } \epsilon_1 \overset{iid}{\sim} \mathcal{N}(0, \sigma_1^2). \tag{1}$$

have been estimated from 1984-2009 data displayed on Figure S11 and resumed in Table S3. As in Chassot et al. (2009), the residual natural mortality at last age (asymptote ς_t in Equ. E9 in BCDH) is connected to the cod condition at year t through a decreasing linear function obtained in [15,16] relating natural mortality to cod condition :

$$\vartheta_t = \gamma_1 - \gamma_2\mathcal{V}_t, \tag{2}$$

where the parameters (γ_1, γ_2) have been set according to extreme measured values of low (0.66) or high (0.9) condition, assuming high (0.38) and low (0.19) asymptotic mortality rates, respectively (cf. Table S3 for the resulting values of (γ_1, γ_2)). To increase realism we add to (2) a centered Gaussian noise with standard deviation 0.04, similar to this which was estimated for the period 1984-2006 [16]. Including the 2007-2009 data did not significantly modify this estimation.

A consequence is that the cod condition-at-age $\mathcal{V}_{a,t}$, given by

$$\mathcal{V}_{a,t} = A\eta_a\mathcal{V}_t, \tag{3}$$

where $\boldsymbol{\eta} = (\eta_1, \ldots, \eta_A)$ (summing to 1, thus implying Equ. BCDH.N3 and $\mathrm{E}[\mathcal{V}_{a,t}] = \mathcal{V}_t$) is the condition proportion-at-age, is a *doubly* random variable. Indeed, these proportions are themselves considered random and following a Dirichlet distribution (Equ. BCDH.N3, *right*).

This second effect leads actually to modify the fecundity-at-age $f_{a,t}$, which has a direct impact on the TEP (Equ. BCDH.E3) then on the recruitment. Indeed, $f_{a,t}$ can be predicted using the log-linear deterministic model proposed by Lambert and Dutil [3, 17] :

$$\log f_{a,t} = \lambda_1 + \lambda_2 \mathcal{V}_{a,t} + \lambda_3 L_{a,t} \quad (4)$$

where $L_{a,t}$ refers to the cod length-at-age. Values for these parameters are resumed in Table S3. Prospective length-at-age values (assuming no dependence in t) were chosen as outputs of an average age-length key given in Table S4, build from averaged values between years 2002 and 2009. Similarly, we used averages on these years to fix prospective values for the proportions of maturing females and the sex ratio.

Finally, the variations of $\mathcal{V}_t$ impact the random variations of the prospective cod weights-at-age $W_{a,t}$. Indeed, we consider the *allometric* length-weight (cm/kgs) relationship $W_{a,t} = \Lambda_t L^3_{a,t}$ where (Equ. BCDH.N4)

$$\Lambda_t = \kappa_1 + \kappa_2 \mathcal{V}_t + \epsilon_2 \quad \text{with } \epsilon_2 \overset{iid}{\sim} \mathcal{N}(0, \sigma_2^2). \quad (5)$$

Values for these parameters have been estimated by regression from data given in Lambert and Dutil (1997) and displayed on Figure S12. Estimations are resumed in Table S3.

Table S3. Supplementary input parameters used in the SIMCAB propagation model. Estimates of parameters λ_i $(i = 1, \ldots, 3)$ are given for lengths-at-age $L_{a,t}$ considered in centimeters.

Notation	Definition	Origin	Value
ν_1	Intercept of linear regression $\mathcal{V}_t \sim$ CIL	Estim.	0.7810
ν_2	Slope of linear regression $\mathcal{V}_t \sim$ CIL	Estim.	0.1009
σ_1	Regression st.dev. of $\mathcal{V}_t \sim$ CIL	Estim.	0.0382
κ_1	Intercept of linear regression $\Lambda_t \sim \mathcal{V}_t$	Estim.	5.967e-13.8
κ_2	Slope of linear regression $\Lambda_t \sim \mathcal{V}_t$	Estim.	4.038e-13.8
σ_2	Regression st.dev. of $\Lambda_t \sim \mathcal{V}_t$	Estim.	2.88e-16.1
λ_1	Intercept of linear regression $\log f_{a,t} \sim (\mathcal{V}_{a,t}, L_{a,t})$	Lambert and Dutil (1997)	-14.525
λ_2	Slope 1 of linear regression $\log f_{a,t} \sim (\mathcal{V}_{a,t}, L_{a,t})$	*ibid.*	1.510
λ_2	Slope 2 of linear regression $\log f_{a,t} \sim (\mathcal{V}_{a,t}, L_{a,t})$	*ibid.*	3.630
γ_1	Intercept of linear regression $\vartheta_t \sim \mathcal{V}_t$	Estim.	0.9025
$-\gamma_2$	Slope of linear regression $\vartheta_t \sim \mathcal{V}_t$	Estim.	-0.7916

Table S4. Prospective cod age-mean-length key based on observations averaged on years 2002-2009.

age	length (cm)	age	length (cm)	age	length (cm)
< 0	0	4	39.4	9	60.0
0	10.1	5	45.9	10	64.6
1	16.8	6	50.4	11	67.0
2	25.0	7	54.6	12	67.4
3	32.4	8	58.5	$>= 13$	68.3

Supplement S5. Simulation technique for prospective fishing rates

In the projection model, the total allowable catch (TAC) is simulated at year $t+1$ using a HCR rule, ie. one can write

$$\mathrm{TAC}_{t+1} \quad = \quad \mathrm{HCR}\left(\mathrm{SSB}_t, B_{\mathrm{lim}}, B_{rec}\right). \tag{6}$$

Assuming the TAC is fully respected each year, the HCR has the effect to fix the yearly fishing rate, which is evaluated by solving the Baranov equation (using a Newton-Raphson minimization routine)

$$F_{t+1} \quad = \quad \arg\min_x \left[\mathrm{TAC}_{t+1} - x \sum_{a=1}^{A} W_{a,t+1} \frac{\varsigma^2_{a,c} N_{a,t+1}\{1-\exp(-M_{a,t+1} - x\varsigma^2_{a,c})\}}{x\varsigma^2_{a,c} + M_{a,t+1}} \right]$$

where $\varsigma^2_{a,c}$ is the commercial selectivity estimated for years after 1993.

References

1. Chassot E, Duplisea D, Hammill M, Caskenette A, Bousquet N, et al. (2009) Role of predation by harp seals *Pagophilus groenlandicus* in the collapse and non-recovery of northern Gulf of St. Lawrence cod *Gadus morhua*. Marine Ecology Progress Series 379: 279–297.

2. Aikake H (1974) A new look at the statistical model identification. IEEE Transactions in Automatic Control 19: 719–723.

3. Lambert Y (2011) Environmental and fishing limitations to the rebuilding of the northern Gulf of St. Lawrence cod stock (*Gadus morhua*). Canadian Journal of Fisheries and Aquatic Sciences 68: 618-631.

4. Mohn R (1999) The retrospective problem in sequential population analysis: an investigation using cod fishery and simulated data. ICES Journal of Marine Science 56: 473-488.

5. DFO (2010) Assessment of the cod stock in the northern Gulf of St Lawrence (3Pn,4Rs) in 2009. Technical report, Canadian Science Advisory Secretariat. Fisheries and Oceans Canada. CSAS 2010/011 Science Advisory Report.

6. DFO (2010) Assessment of the northern Gulf Gulf of St Lawrence (3Pn,4Rs) cod stock in 2011. Technical report, Canadian Science Advisory Secretariat. Fisheries and Oceans Canada. CSAS 2012/005 Science Advisory Report.

7. Hammill M, Stenson G, Doniol-Valcroze T, Mosnier A (2011) Northwest Atlantic Harp Seals Population Trends, 1952-2012. Technical report, Canadian Science Advisory Secretariat. Fisheries and Oceans Canada. CSAS 2011/nnn.

8. Galbraith P, Chassé J, Gilbert D, Larouche P, Brickman D, et al. (2011) Physical Oceanographic Conditions in the Gulf of St. Lawrence in 2010. Technical report, Canadian Science Advisory Secretariat. Fisheries and Oceans Canada. CSAS 2011/045 Research Document.

9. DFO (2011) Historical Grey Seal Abundance and Changes in the Abundance of Grey Seal Predators in the Northwest Atlantic. Technical report, Canadian Science Advisory Secretariat. Fisheries and Oceans Canada. CSAS 2011/0026 Science Advisory Report.

10. Haddon M (2001) Modelling and Quantitative Methods in Fisheries. Chapman & Hall, – pp.

11. Dupuis J (2002) Estimation bayésienne d'un modèle multi-états markovien *in French*): in *Méthodes Bayésiennes en Statistique*, Technip.

12. Virtala M, Kuikka S, Arjas E (1998) Stochastic virtual population analysis. ICES Journal of Marine Science 55: 892–904.

13. Follestad T (2003) Stochastic modelling and simulation based inference of fish population dynamics and spatial variation in disease risk. Ph.D. thesis, Norwegian University of Science and Technology.

14. Minto C, Myers RA, Blanchard W (2008) Survival variability and population density in fish populations. Nature 452: 344–347.

15. Dutil JD, Lambert Y (2000) Natural mortality from poor condition in atlantic cod (Gadus morhua). Canadian Journal of Fisheries and Aquatic Sciences 57: 826–836.

16. Chassot E, Caskenette A, Duplisea D, Hammill M, Bourdages H, et al. (2007) A model of predation by harp seals (*Phoca groenlandica*) on the northern Gulf of St. Lawrence stock of Atlantic cod (*Gadus morhua*). Technical report, Canadian Science Advisory Secretariat. Fisheries and Oceans Canada. CSAS 2007/066.

17. Lambert Y, Dutil JD (1997) Condition and energy reserves of Atlantic cod (*Gadus morhua*) during the collapse of the northern Gulf of St. Lawrence stock. Canadian Journal of Fisheries and Aquatic Sciences 54: 2388–2400.

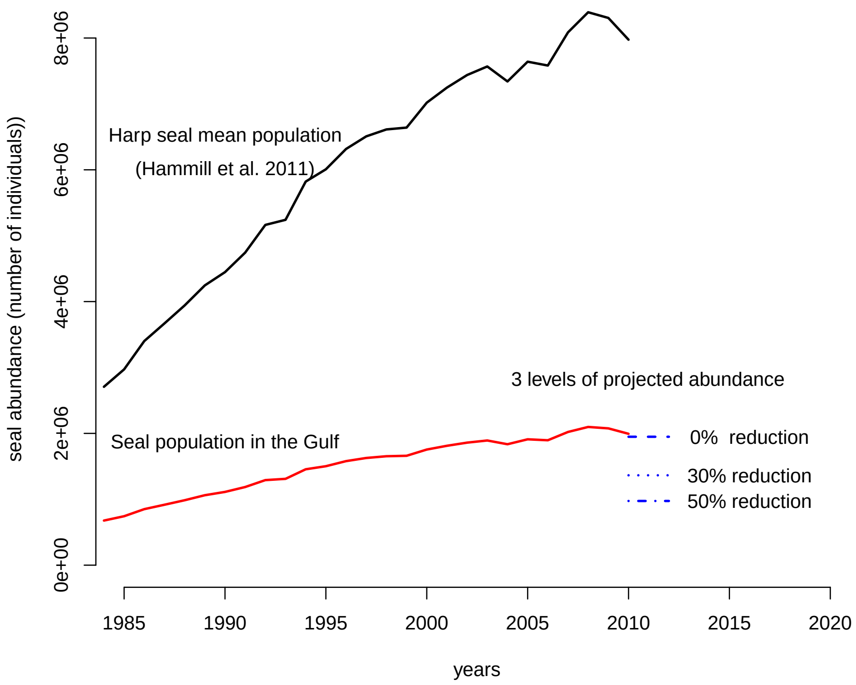

Figure S1. Average levels of estimated harp seal abundance in the Northwest Atlantic [7] and specific to the Gulf, and three plausible levels of mean abundance used for the projection study.

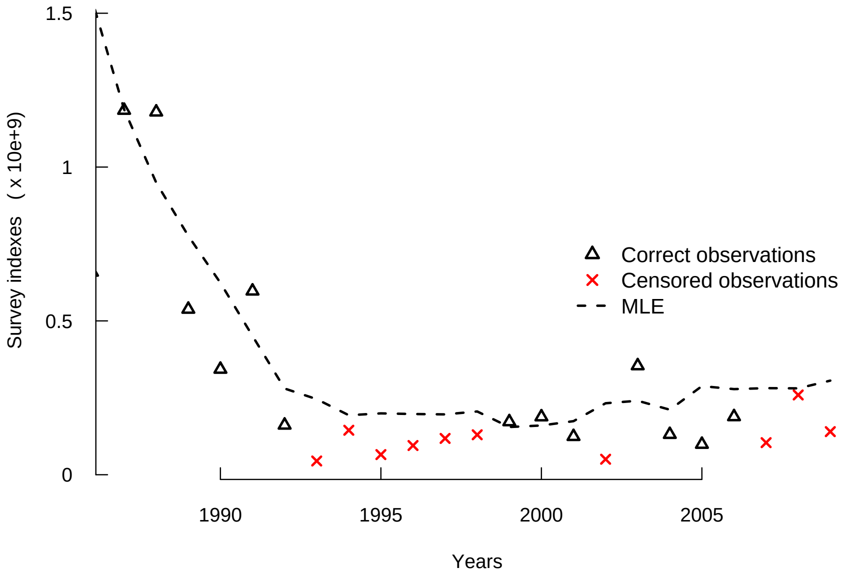

Figure S2. Observed and estimated survey indexes by the SIMCAB model. Censored observations correspond to partial sums over ages of indices-at-age, not including missing values.

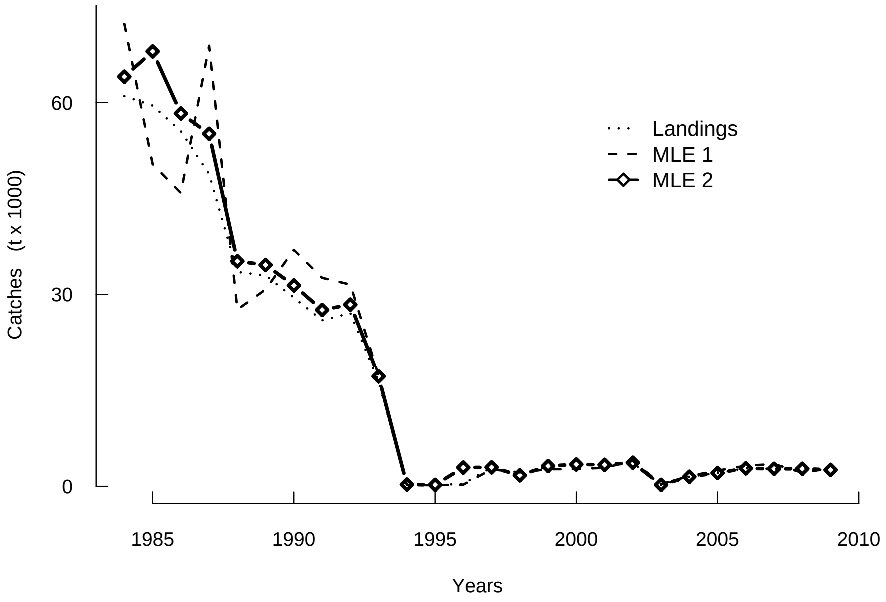

Figure S3. Landings and SIMCAB catch estimates. MLE = maximum likelihood estimate; MLE 1 is given by the first version of the model (Chassot et al. 2009); MLE 2 = present study.

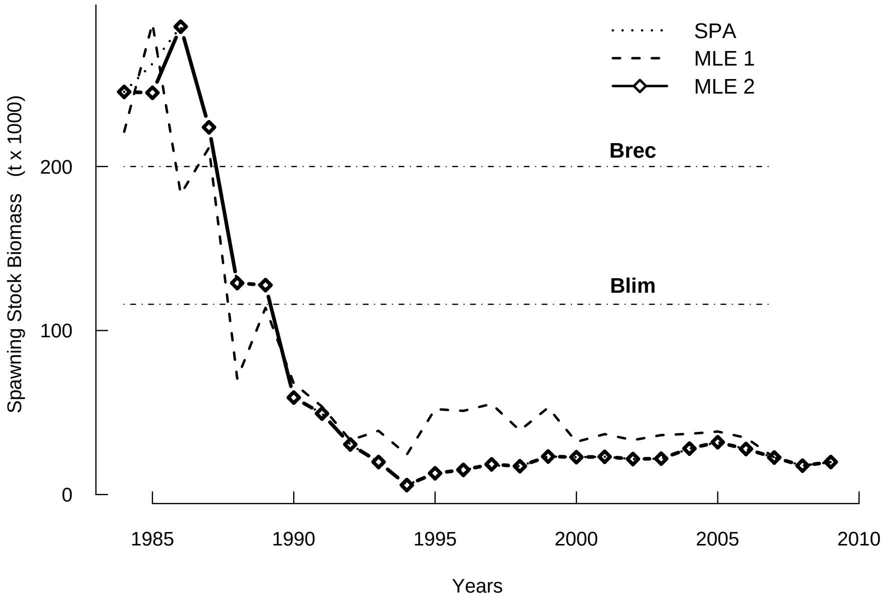

Figure S4. Spawning stock biomass (SSB) estimates for the cod stock of the northern Gulf of St. Lawrence derived from sequential population analysis (SPA; DFO 2010) and from the SIMCAB estimation model. B_{lim} and B_{rec} are the limit and recovery biological reference points, respectively. MLE = maximum likelihood estimate; MLE 1 = Chassot et al. 2009; MLE 2 = present study.

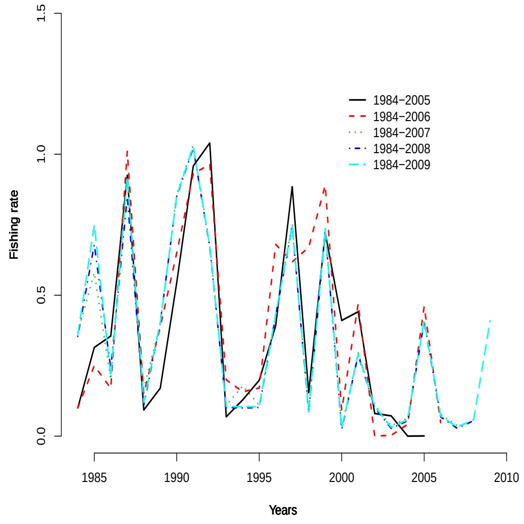

Figure S5. Estimates of fishing rates from retrospective analyses.

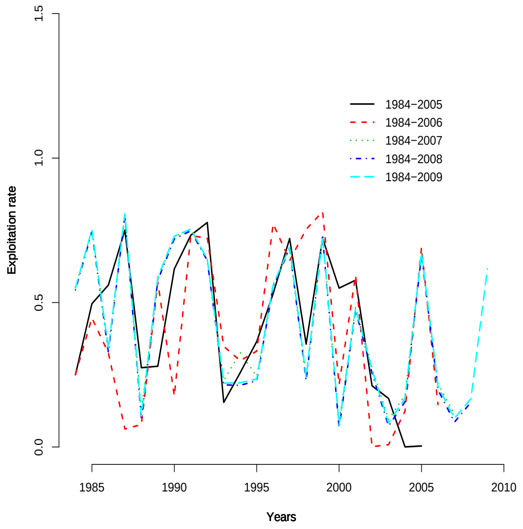

Figure S6. Estimates of exploitation rates from retrospective analyses.

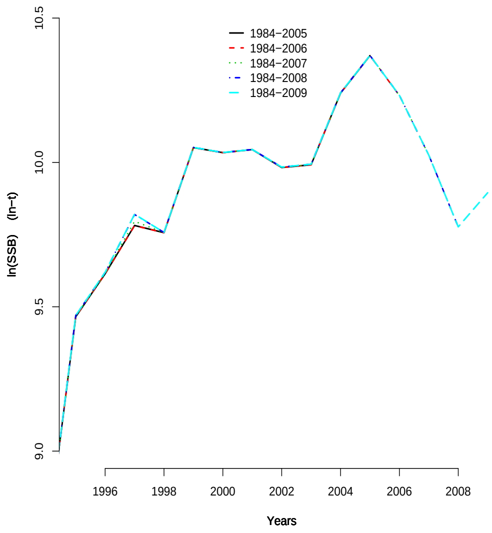

Figure S7. Estimates of ln SSB from retrospective analyses.

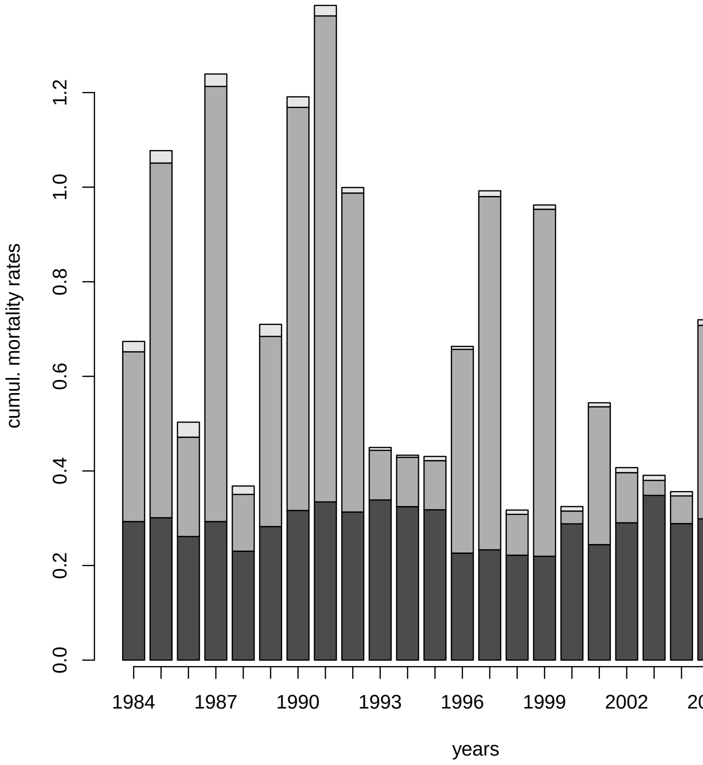

Figure S8. Cumulative annual mortality rates for cod. Dark grey: natural (other than seal predation) mortality for spawners; light grey: fishing mortality; white: predation mortality (ages 1-4).

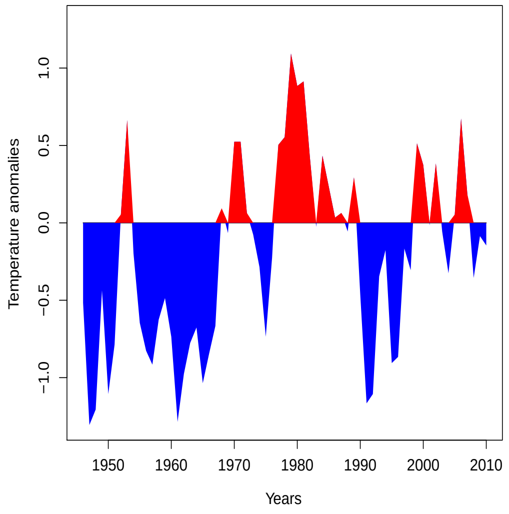

Figure S9. Overview of anomalities with respect to the average CIL temperature (in ˚C).

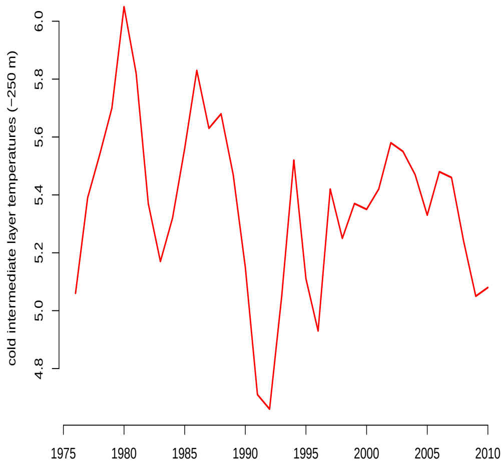

Figure S10. Annual temperatures of the cold intermediate layer (-250 m, in ˚C) (courtesy of P.S. Galbraith, cf. [8]). Only the period 1989-2009 was retained for fitting the linear model linking cod condition and water temperature (Equ. (1) in this document).

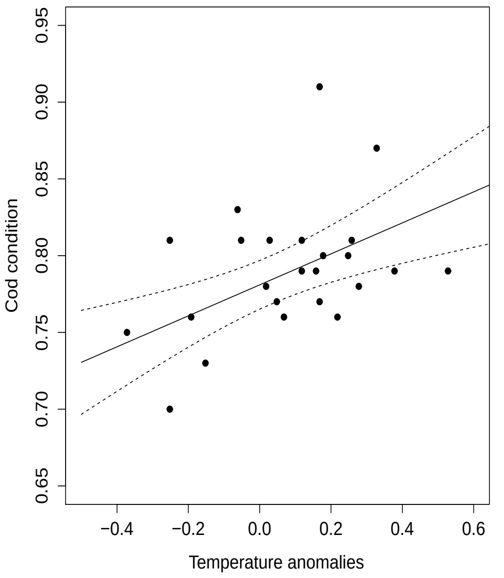

Figure S11. Linear regression of $\mathcal{V}_t \sim$ CIL. The adjusted Pearson's coefficient $r = 0.58$, with $p-$value < 0.01.

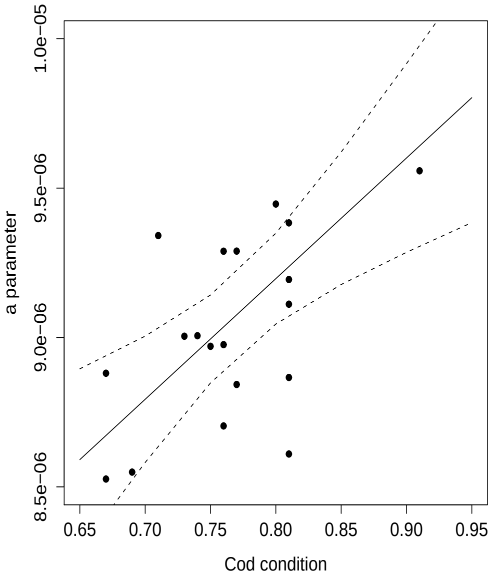

Figure S12. Linear regression of $\Lambda_t \sim \mathcal{V}_t$.

Table S5. $P-$values of survey proportions-at-age with respect to their predicted (propagated) distribution, calibrated by maximum likelihood estimation over the previous years. Columns (a), (b) and (c) represent age classes 1-4, 5-8 and 9-13. Results are averaged on these classes (standard deviations are given between parentheses).

Years of projection	Years of estimation					
	**1984-2005**			**1984-2006**		
	(a)	(b)	(c)	(a)	(b)	(c)
2006	0.481 (0.08)	0.522 (0.11)	0.479 (0.09)			
2007	0.497 (0.12)	0.445 (0.10)	0.514 (0.10)	0.426 (0.11)	0.398 (0.12)	0.477 (0.09)
2008	0.382 (0.14)	0.504 (0.09)	0.532 (0.07)	0.448 (0.08)	0.601 (0.08)	0.571 (0.10)
2009	0.581 (0.13)	0.522 (0.06)	0.495 (0.11)	0.462 (0.08)	0.565 (0.04)	0.514 (0.07)
	**1984-2007**			**1984-2008**		
	(a)	(b)	(c)	(a)	(b)	(c)
2008	0.387 (0.13)	0.455 (0.09)	0.394 (0.15)			
2009	0.457 (0.10)	0.509 (0.05)	0.611 (0.09)	0.394 (0.12)	0.491 (0.08)	0.556 (0.08)

Table S6. $P-$values of catch proportions-at-age with respect to their predicted (propagated) distribution, calibrated by maximum likelihood estimation over the previous years. Columns (b) and (c) represent age classes 5-8 and 9-13. Results are averaged on these classes (standard deviations are given between parentheses).

Years of projection	Years of estimation			
	**1984-2005**		**1984-2006**	
	(b)	(c)	(b)	(c)
2006	0.612 (0.13)	0.477 (0.11)		
2007	0.542 (0.06)	0.549 (0.05)	0.426 (0.10)	0.538 (0.06)
2008	0.597 (0.10)	0.488 (0.09)	0.384 (0.17)	0.427 (0.15)
2009	0.469 (0.11)	0.534 (0.12)	0.607 (0.09)	0.511 (0.06)
	**1984-2007**		**1984-2008**	
	(b)	(c)	(b)	(c)
2008	0.391 (0.16)	0.417 (0.14)		
2009	0.472 (0.11)	0.509 (0.07)	0.466 (0.10)	0.535 (0.07)
